# Forecasting stress transitions using ecological momentary assessment data and machine learning

**DOI:** 10.1016/j.invent.2026.100969

**Published:** 2026-07-01

**Authors:** Rutger van der Linden, Diana Burychka, Asmae Doukani, Gonçalo Gonçalves, Eline Henrotte, Rocio Herrero, Milena Imwinkelried, Elona Krasniqi, Samuel Lam, Lisa Groenberg Riisager, Kathrin Schopf, Claire Rosalie van Genugten, Minja Westerlund, Rosa Baños, Arlinda Cerga Pashoja, Naim Fanaj, Tobias Krieger, Kim Mathiasen, Artur Rocha, Silvia Schneider, Andre Sourander, Annet Kleiboer, Mark Hoogendoorn, Aneta Lisowska

**Affiliations:** a Department of Computer Science, Vrije Universiteit Amsterdam, Amsterdam, The Netherlands; b Centre for Urban Mental Health, University of Amsterdam, Amsterdam, The Netherlands; c Cancer Center Amsterdam, VU University Medical Center (VUmc), Amsterdam, The Netherlands; d Clinical, Neuro-, and Developmental Psychology, Vrije Universiteit Amsterdam, Amsterdam, The Netherlands; e Amsterdam Public Health, Mental Health, Amsterdam, The Netherlands; f St Mary’s University of Twickenham, London, England, United Kingdom; g Department of Psychology, University for Business and Technology, Prishtina, Kosovo; h Alma Mater Europaea Campus College Rezonanca, Prishtina, Kosovo; i PMSH, Prizren, Kosovo; j Department of Psychology and Behavioural Sciences, School of Business and Social Sciences, Aarhus University, Aarhus, Denmark; k Research Centre for Child Psychiatry, University of Turku, Turku, Finland; l INVEST Research Flagship Centre, University of Turku, Turku, Finland; m Department of Child Psychiatry, Turku University Hospital, Turku, Finland; n Polibienestar Research Institute, Universitat de Valencia, Valencia, Spain; o Department of Personality, Evaluation and Psychological Treatment, Faculty of Psychology, Universitat de Valencia, Valencia, Spain; p CIBER of Physiopathology of Obesity and Nutrition (CIBEROBN), Instituto de Salud Carlos III, Madrid, Spain; q Department of Basic and Clinical Psychology and Psychobiology, Universitat Jaume I, Castellón, Spain; r Department of Clinical Child and Adolescent Psychology, Mental Health Research and Treatment Center, Faculty of Psychology, Ruhr University Bochum, Bochum, Germany; s Institute of Psychology, University of Bern, Bern, Switzerland; t Institute for Systems and Computer Engineering, Technology and Science (INESC TEC), Porto, Portugal

**Keywords:** Machine learning, Ecological momentary assessment (EMA), Stress, Just-in-time adaptive intervention (JITAI), mHealth

## Abstract

Stress is associated with many negative effects, including inadequate sleep, reduced learning and memory, and a higher risk of mental health conditions. Given these effects, it is important to explore effective strategies for stress management and intervention. One promising approach is the use of ecological momentary assessments (EMAs), which allow us to measure an individuals’ experiences in their natural environments, offering valuable data to inform just-in-time adaptive interventions (JITAIs). Machine learning can further enhance JITAIs by forecasting stress-related emotional states, enabling proactive intervention delivery to prevent heightened stress. In this study, we focus on forecasting stress utilizing data from a large mental health project. During this project, EMA data was collected from different vulnerable target groups across Europe, including youth, older adults, migrants, and individuals with low socioeconomic status. We formulated the forecasting task as a binary classification problem: predicting either transitions from normal to elevated stress or the stability of normal stress, based on a person’s stress distribution. This approach simplifies the task, supports personalized predictions, and enables actionable insights, as predicting elevated stress can directly trigger support. Our results demonstrate that machine learning models are capable of forecasting stress transitions (ROC-AUC = 0.70 vs. 0.50 for a random classifier), although predicting transitions to elevated stress proved more challenging than identifying stable normal stress. Models trained on combined data from all populations performed comparable to those trained on individual populations. Furthermore, cross-country evaluations indicated that population-specific models generalized well across most populations.

## Highlights

• ML models can forecast stress level transitions using only EMA data.
• Forecasting formulation supports proactive, just-in-time adaptive interventions.
• ML model trained on multi-population data generalizes across diverse target groups.

## 1 Introduction

Stress can have a significant impact on health (O’Connor et al., 2021). It is associated with an increased risk of conditions such as anxiety, depression, and substance abuse (Konstantopoulou et al., 2020, Sinha et al., 2024). Additionally, it has been shown to affect learning and memory and contribute to unhealthy daily habits, including inadequate sleep and reduced motivation for physical activity (Geiker et al., 2018, Kim and Kim, 2023). Due to these effects, identifying effective strategies for stress management is essential.

One promising approach to managing stress and collecting mental health data is the use of mobile health (mHealth), which involves mobile and wireless technologies (e.g., smartphones and wearables) to support health care practices (Istepanian, 2022). A key advantage of mHealth is its ability to support ecological momentary assessments (EMAs), which consists of repeatedly sampling individuals’ current behaviors and experiences in their natural environments (Balaskas et al., 2021, Shiffman et al., 2008). EMA data, based on the monitoring of dynamic psychological states (e.g., stress) in real time, can be used to inform the timing and content of personalized interventions. These interventions, known as just-in-time adaptive interventions (JITAIs) (van Genugten et al., 2025, Nahum-Shani et al., 2015, Schneider et al., 2024), are designed to help at moments of increased vulnerability or increased receptivity to positive behavior changes (Hsu et al., 2025, Nahum-Shani et al., 2016).

Machine learning (ML) techniques offer the potential to further tailor the delivery of (stress-targeting) JITAIs (van Genugten et al., 2025). In particular, ML can be used to forecast changes in stress, enabling interventions to be delivered during periods of heightened vulnerability. Such predictive strategies may help prevent declines in well-being (Mikus et al., 2018). Although previous studies have shown that stress can be predicted from EMA data using ML approaches (Li et al., 2022, Ng et al., 2022), this work has primarily focused on predicting exact stress levels or classifying stress based on predefined thresholds. As a result, the implications of these predictions for intervention delivery remain largely unexplored. In addition, limited attention has been paid to the transferability of models across populations, despite its importance for real-world implementation. Examining both population-specific models and models trained on pooled data from multiple populations is therefore critical for assessing generalizability and informing which models are most suitable for deployment.

In this work, we focus on stress forecasting specified as a binary classification task, namely, predicting a transition from normal stress to elevated stress or the stability of normal stress. To account for individual differences in stress perception (Harris et al., 2023), we define normal stress and elevated stress based on each participant’s stress distribution over the past 7 days. This approach simplifies the task, supports personalized predictions, and enables timely and actionable insights, as predicting an increase in stress can directly trigger intervention (e.g., brief exercises or a notification), although no interventions are triggered in the present study.

For this, EMA data from the RECONNECTED project (Project No. : 101081020) is used, in which mental health information from vulnerable populations across European countries is collected. During the study, participants completed a set of EMA items administered up to five times a day (see Table A.1 in Appendix A). Using this data, we evaluate prediction performance and compare models trained on combined data from all populations with models trained separately for each country. We further assess cross-country performance by testing models trained on one country’s data on another country’s population. Additionally, we examine whether using only stress-related features yields similar performance to using all EMA items, thereby potentially reducing EMA burden. Finally, we employ SHAP feature importance analysis (Lundberg and Lee, 2017) to identify the most influential predictors and interpret their impact on model outputs.

Our contributions are as follows:

• Development of an EMA-based ML model to predict transitions from normal stress to elevated stress.
• Cross-country evaluation to assess the effect of distribution shifts.
• Evaluation of models trained with light-weight features versus models using all EMA questions to determine whether EMA burden can be reduced.
• Feature importance analysis to identify the most influential features and their effect on model predictions.

## 2 Related work

Several studies have explored the application of ML for predicting EMA data, including self-reported mood (Mikus et al., 2018), depressive symptoms (De la Barrera et al., 2024), and stress (Li et al., 2022, Ng et al., 2022). For instance, De la Barrera et al. (2024) applied ML timeseries models, such as recurrent neural networks, to predict mood values, whereas De la Barrera et al. (2024) employed regression techniques, including logistic regression and random forests, to forecast depressive symptoms. In the context of stress prediction, Ng et al. (2022) focused on next-day categorical stress prediction in pregnant women using six widely applied ML models, including random forests and adaptive boosting, and demonstrated that current stress is an important predictor of next-day perceived stress for their ML model. In contrast, Li et al. (2022) predicted both exact and categorized stress levels over multiple days in a student population during the COVID-19 pandemic, employing techniques such as gradient boosting and support vector regression. Collectively, these studies demonstrate the feasibility of applying ML techniques to predict mental health outcomes from EMA data. Compared with prior work, our study incorporates personalized thresholds for defining stress, an important consideration given that individuals experience stress differently (Harris et al., 2023). Moreover, we evaluate both population-specific models and models trained on data from multiple populations. This enables insights into model generalizability and helps determine which models are most suitable for deployment.

A substantial body of research has explored the use of wearable sensor data combined with ML to detect stress in real time, often presented as a possible basis to trigger intervention (Khan et al., 2024, Lisowska et al., 2021, Sarker et al., 2017). Sensors in these systems commonly include accelerometers, gyroscopes, magnetometers, blood volume pulse monitors, electrocardiograph sensors, and respiration sensors (Khan et al., 2024, Lisowska et al., 2021, Sarker et al., 2017). A particularly relevant study in this context is by Bögemann et al. (2026), who implemented a stress detection system. In their approach, ML was used to assess the reliability of incoming physiological data within a feature extraction algorithm (Bögemann et al., 2026), whereas other studies apply ML more directly for detecting current stress (Khan et al., 2024, Lisowska et al., 2021, Sarker et al., 2017) Unlike many previous studies (Khan et al., 2024, Lisowska et al., 2021, Sarker et al., 2017), their algorithm was tested in a real-life application to trigger interventions (Bögemann et al., 2026). The JITAI algorithm combined features from EMAs and ecological physiological assessments to generate a composite stress score. Interventions were triggered when this score exceeded an individually defined stress threshold. The feasibility of their approach was demonstrated through technical implementation, participant adherence, and user experience. However, their work (Bögemann et al., 2026) and other related work (Khan et al., 2024, Lisowska et al., 2021, Sarker et al., 2017) focused on detecting current stress rather than predicting future stress, whereas the latter provides insights for interventions aimed at preventing future declines in well-being (Mikus et al., 2018).

## 3 Methods

### 3.1 Dataset

#### 3.1.1 Study design

The dataset was obtained from a two-week EMA study conducted as part of the RECONNECTED project (OSF registration: https://doi.org/10.17605/OSF.IO/KTJ7Q). The RECONNECTED project aims to better understand how multiple global developments (e.g., climate change, digitalization, and a changing population) affect the mental health of European citizens. The study recruited participants from nine countries: the United Kingdom (UK), France, Kosovo, Switzerland, Germany, the Netherlands, Denmark, Finland, and Spain. Data from the France sample was not utilized as insufficient data was available at the time of extraction.

Each study site focused on a specific vulnerable group: in the UK, these were non-western migrants aged 18 or older; in Germany, children aged 12–16 in secondary education in North-Rhine Westphalia; in Kosovo, adults aged 18 or older residing in low socioeconomic status (SES) neighborhoods; in Switzerland, German-speaking adults aged 55 or older from the general population; in Denmark, youth aged 16–25; in the Netherlands, older adults aged 55 or older in urban areas; in Spain migrants from Latin America aged 18 or older; and in Finland, young adults aged 18–20 from the general population.

#### 3.1.2 Recruitment and reimbursement

Recruitment methods varied across study sites and included approaches such as posters at educational institutions, social media posts, and flyers in community centers. A site-specific website contained all information about study participation. Individuals who wanted to join the study submitted their contact details and completed a short questionnaire for eligibility screening. After providing informed consent, participants were invited to complete a baseline survey before beginning 14 days of EMA. After the study, participants receive a reimbursement. The specific form of compensation and the amount were determined by each study site separately. A full overview of the recruitment and reimbursement strategy is shown in Table B.1 in Appendix B.

#### 3.1.3 Procedure

Participants were asked to complete five smartphone-based EMA measurements per day: one in the morning (08:00–10:00), three during the daytime (10:00–13:00, 13:00–16:00, and 16:00–19:00), and one in the evening (19:00–21:00). This prompt frequency is commonly used in EMA studies (Williams et al., 2021) and was chosen to balance participant burden while gathering sufficient information. The schedule was also designed to align with participants’ daily schedule by distributing prompts across periods when most individuals are typically awake.

Within each window, a prompt was sent at a random time in the first 30 min; if no response was recorded, a second prompt followed in the final 30 min of the window, with an offset of 10 min to avoid receiving notifications too close to each other. Participants were instructed to complete the EMAs as soon as possible after receiving the prompt (notification on their smartphone). However, the notification will remain visible for two hours beyond the time slot. A shorter timeframe for completing the EMAs was not set as not all participants can respond immediately due to contextual factors. Although participants did not receive formal training on completing EMA measurements, they were provided with instructions on how to complete them.

#### 3.1.4 EMA measures

A central concept in the RECONNECTED project is resilience, defined as the process of maintaining mental health or recovering rapidly when exposed to significant stressors or challenging life events that require adaptation (Kalisch et al., 2017, Richardson, 2002). Resilience processes substantially shape how individuals respond to change such as those caused by societal challenges (Schäfer et al., 2024).

EMA items were selected to capture mechanisms identified through a review of evidence-based resilience interventions (Boniwell et al., 2023, Chesak et al., 2015, Riggle et al., 2014, Werneburg et al., 2018). These mechanisms include stress anticipation, stressful events and their impact, positive and negative affect, emotion regulation and received social support. Additionally, stressors and adverse situations are measured to evaluate stress resistance. Physical activity and sleep quality were also assessed (Arora et al., 2022).

Affect items (e.g., “At the moment I feel…sad/stressed/relaxed/energetic”) were based on the Hedonic and Arousal Affect Scale (HAAS; Roca et al. (2023)) design. Participants were also prompted about their emotion regulation abilities, providing insights into existing coping skills, specific emotion regulation strategies, and maladaptive use (Polizzi and Lynn, 2021).

EMA items were translated from English into the local language of each study site using a forward–backward translation procedure, in which the back-translated version was compared with the original item to ensure consistency. Most items used a 7-point Likert scale, with responses entered via an on-screen slider (1 = not at all, 7 = extremely). Verbal anchors were displayed to participants. Stress was assessed at each prompt using the item “At the moment I feel stressed”. EMA items have not been validated for this study. Demographic information on age and gender was also collected. A full overview of EMA items, response options, and prompt timing is provided in Table A.1 of Appendix A. Additionally, only non-conditional items were included in the analyses, meaning items whose presentation did not depend on responses to other EMA items.

#### 3.1.5 Population statistics

Summary statistics for each country’s sample are presented in Table 1, including only participants who had completed at least one EMA questionnaire. The total number of participants was 2291 and their total combined sample size was 158284. Missing stress observations refer to prompts at which a participant did not complete the stress EMA measurement. Floor effects and the intraclass correlation coefficient (ICC) are reported in the results section to assess stress response distributions and the relative contribution of within- versus between-person variability in stress measurements (Siepe et al., 2025). For calculating the ICC, python package Pingouin was used (Vallat, 2018).

**Table 1:** Population statistics.

Country	Group	Number of participants (N)	Number of responses (n)	Age (mean (SD))	Gender (Female)	Stress (mean (SD))^b^	Missing stress
*Denmark*	Youth	344	23 776	22.19 (2.21)	296 (86.05%)	2.18 (1.42)	4951 (20.82%)
*Finland*	Youth	237	16 009	19.82 (2.18)	200 (84.39%)	2.40 (1.41)	2421 (15.12%)
*Germany*	Youth	346	24 220	14.34 (1.57)	186 (53.76%)	2.47 (1.68)	7297 (30.13%)
*Kosovo*	Low SES^a^	339	23 730	27.43 (11.16)	277 (81.71%)	2.21 (1.56)	5546 (23.37%)
*Netherlands*	Older adults	152	10 523	65.18 (6.84)	114 (75.00%)	1.81 (1.17)	2000 (19.01%)
*Spain*	Latin American migrants	248	16 276	36.27 (10.79)	187 (75.40%)	2.08 (1.43)	3344 (20.55%)
*Switzerland*	Older adults	357	24 990	65.80 (6.85)	219 (61.34%)	1.64 (1.07)	2670 (10.68%)
*UK*	Non-western migrants	268	18 760	29.09 (8.15)	119 (44.40%)	2.96 (1.69)	2732 (14.56%)

**Total**	–	**2291**	**158 284**	**33.51 (19.56)**	**1598 (69.75%)**	**2.21 (1.50)**	**30961 (19.56%)**

a SES refers to socioeconomic status.

b Stress ranges from 1 to 7.

### 3.2 Stress measurement

In this study, we specifically focused on self-reported stress, reflecting an individual’s perceived experience of stress in daily life (Vaessen et al., 2021). We do not examine physiological stress responses, such as autonomic nervous system (ANS) activity reflected in heart rate, blood pressure, or other wearable sensor measurements (Rodrigues et al., 2015). We define stress as subjective stress reported through the EMA item: “At the moment, I feel stressed”, rated on a 7-point Likert scale (1–7). This measure reflects participants’ immediate perceived stress at the time of assessment and is used to define the prediction target for the machine learning models.

### 3.3 Target variable

#### 3.3.1 Task formulation

In this study, we conceptualized stress forecasting as a binary prediction task: forecasting either (1) transitions from normal to elevated stress or (2) the stability of normal stress, based on a person’s stress distribution. This formulation was used because it simplifies the task, allows for personalized predictions, and allows direct triggering of JITAIs. This personalized definition emphasizes within-person deviations from typical stress levels rather than between-person differences in absolute stress levels. We chose to frame the prediction task specifically as the transition from a normal to an elevated stress state since the intended goal is to support the triggering of a JITAI at the moment when an increase in stress is predicted. The goal of such a JITAI would be to foster individuals when an increase in stress is expected.

#### 3.3.2 Personalized stress threshold

Since stress experiences vary across individuals (Harris et al., 2023), normal stress and elevated stress were defined relative to each person’s stress distribution rather than using a fixed threshold across all participants. To estimate this distribution, a rolling seven-day historical window (past 35 timepoints) was used to provide a stable measure while reducing the influence of outliers. The current timepoint’s stress was not included for defining the personal median stress value. Ratings below or equal to the median stress of a participant were classified as normal stress. A stress value above the median stress of a participant was classified as elevated. A historical time window of seven days was considered appropriate as it also allows to capture the fluctuations which can arise due to time-of-day and weekday effects (Siepe et al., 2025).

#### 3.3.3 Prediction window

Predictions target the next time slot, with windows defined as: morning (08:00–10:00), three midday periods (10:00–13:00, 13:00–16:00, 16:00–19:00), and evening (19:00–21:00). Each prediction therefore looks a few hours ahead within the same day, except for the evening slot, which forecasts a transition into the following day. This distinction also carries clinical relevance, as the evening prediction anticipates the patient’s state at the start of the next day. Given these differences across slots, we report model performance separately for each time window.

#### 3.3.4 Labeling samples

A stress transition was defined as a change from a normal stress state to an elevated stress state. Timepoints at which participants were already in an elevated stress state were excluded, as no new transition to elevated stress could occur from these observations. Including such observations in a machine learning model would serve a different purpose, namely, triggering interventions when continuity of elevated stress is expected, or a decrease from elevated to normal stress. This falls outside the scope of the present paper. When the stress value at the current timepoint was missing, the most recent available stress rating was used to determine whether the participant was in a normal or elevated stress state. This approach preserved temporal continuity in the stress trajectory and allowed predictions to be generated even when the current EMA stress value was unavailable. However, no prediction was generated for observations where the stress value at the subsequent timepoint was missing, as the corresponding target state could not be determined. To illustrate the target variable, Fig. 1 presents stress trajectories for two participants. In this figure, transitions to elevated stress are marked with red dots, while the first seven days are shaded in gray to indicate the minimum historical window required to calculate the rolling median stress value. Gaps between the dots represent timepoints at which participants did not complete a stress EMA assessment.

Fig. 1Stress developments of two participants.Fig. 1

**(a):**
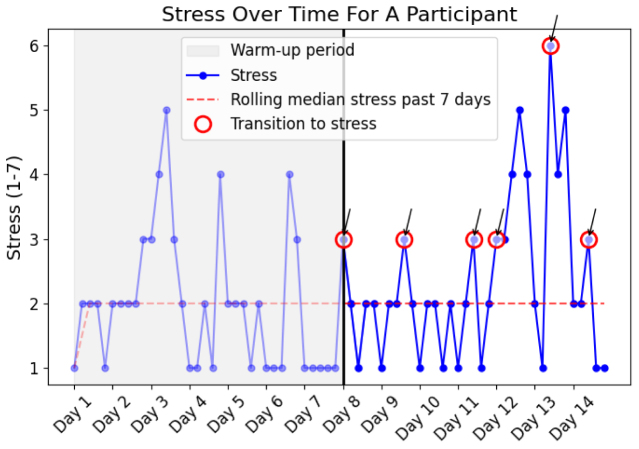
Example participant 1.

**(b):**
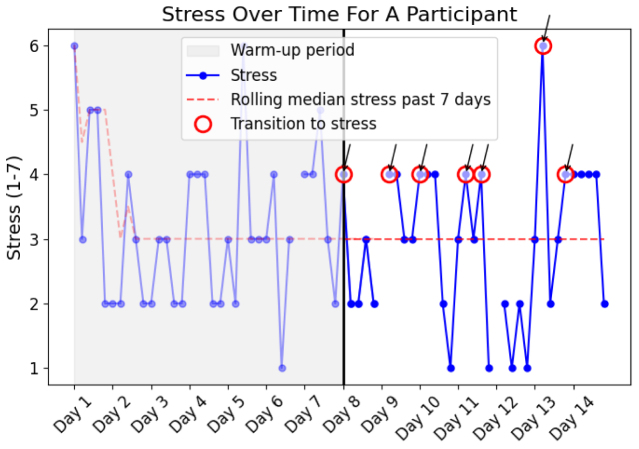
Example participant 2.

### 3.4 Machine learning models

#### 3.4.1 Features and preprocessing

For the EMA questions listed in Table A.1 of Appendix A, a set of features was derived for training the ML models. This was done for both the stress EMA question and the other EMA questions. These features were based on the work of Guo et al. (2020), who identified timeseries summary statistics relevant for clinical prediction tasks with physiological timeseries. Clinical timeseries data share several important characteristics with EMA data, namely, they are temporally ordered, repeatedly sampled, and reflect within-person variability over time. Therefore, comparable summary statistics were considered appropriate for engineering features from EMA timeseries data. Specifically, the following statistics were calculated both on a daily basis and cumulatively over the previous seven days: mean, median, standard deviation, minimum, maximum, range, kurtosis, skewness, and variance. In addition, lagged values and their pairwise differences were calculated to identify trends. These summary statistics were also applied to a feature indicating the proportion of elevated stress over the past timepoints and a feature indicating the proportion of transitions to elevated stress over the past timepoints. Formulas for these features are provided in Appendix C. Timepoints in which participants had a stress state classified as ‘elevated’ were not discarded for calculating features.

Participants whose adherence with the EMA stress rating was less than 50% were excluded. A similar adherence threshold has been used in prior work; for example, De la Barrera et al. (2024) excluded participants who completed fewer than 65% of surveys when training their machine-learning model to predict depressive symptoms. Adherence was calculated based only on observed values and did not include imputed data from carried-forward observations. For model evaluation, the dataset was split at the participant level into a training (70%) and test (30%) set. This was done per country, allowing evaluation of model performance separately for each population.

#### 3.4.2 Model set-Up

ML models were trained with XGBoost (Chen and Guestrin, 2016), and scikit-learn (Pedregosa et al., 2011) random forest and logistic regression. Model tuning was conducted using 5-fold stratified cross-validation on the training set at the participant level, ensuring that the distribution of participants from each country remained consistent across folds. This was achieved by using a country indicator as the stratifying variable, ensuring that the proportion of participants from each country was equal across the training and validation folds during cross-validation.

The hyperparameters optimized for the XGBoost, random forest, and logistic regression models are detailed in respectively Table D.1, Table D.2, and Table D.3 in Appendix D. These hyperparameters were chosen based on cross-validation experiments. Feature selection was performed based on the feature importance scores provided by each model. For the logistic regression models, missing values were first imputed using forward filling per participant. If there were no values for the first timepoints, the median value of the corresponding feature column over all participants was used. Imputation was not needed for the XGBoost and random forest models as they can inherently handle missing data. Imputed values were not used for feature calculation. Forward-filling imputation was applied only after feature statistics had been calculated. We assumed data are missing at random (MAR), meaning that missingness depends on observed variables but not the unobserved data (Heymans and Twisk, 2022). We nonetheless acknowledge that Missing Not At Random (MNAR) cannot be fully excluded. When data is MNAR, missingness is systematically related to the unobserved data (Heymans and Twisk, 2022).

### 3.5 Model configurations

We implemented multiple model configurations to evaluate generalizability across populations and the potential to reduce EMA burden. To evaluate whether EMA burden could be reduced, ML models were trained and evaluated on data from all populations using either features derived from the full EMA question set or only the stress EMA question (“At the moment I feel stressed”). Performance was compared across all ML model types (XGBoost, logistic regression, random forest), and subsequent analyses were conducted using the best-performing model type. We also conducted two ablations: one excluding inference of missing stress values from the most recent prior observation, and one including all four possible prediction targets. To examine whether predictive performance varied across the day, results are shown per time slot. Additionally, performance was also reported across the different personal stress thresholds. Furthermore, to assess how well models generalize across populations, we compared a model trained on the combined training sets of all populations against models trained separately per country. Finally, models trained on one country were evaluated on data from another country, giving insights into cross-country-and-population performance. Code for training the ML models can be found under the following link: https://github.com/Rutgergl/GitHub_TimeseriesForecasting.

### 3.6 Evaluation

#### 3.6.1 Performance measures

The main metric used to evaluate model performance was the ROC–AUC, which was also used for optimizing hyperparameters. This metric reflects how well the model can distinguish between the two prediction classes. In addition, precision, recall, and accuracy are reported. The mean ROC–AUC and the standard deviation is reported for the 5-fold cross-validation on the training set applied during hyperparameter tuning. For acquiring a 95% confidence interval on the test set, performance was estimated using 100 bootstraps, generated by resampling participants with replacement.

#### 3.6.2 Baseline predictors

To provide context for the performance of the ML models, two dummy baseline predictors are reported. This includes ‘most frequent’, which always returns the most frequent class label in the dataset, and ‘stratified’, where the prediction is made by randomly sampling vectors from a multinomial distribution parametrized by the class probabilities (Santana et al., 2020).

#### 3.6.3 Explainability

To understand how features impact the decision-making of the ML models, explainable AI technique SHAP (Lundberg and Lee, 2017) was applied. This method was chosen because it shows the impact of feature values on model outputs and can be applied across different ML algorithms.

## 4 Results

### 4.1 Dataset statistics

**Fig. 2:**
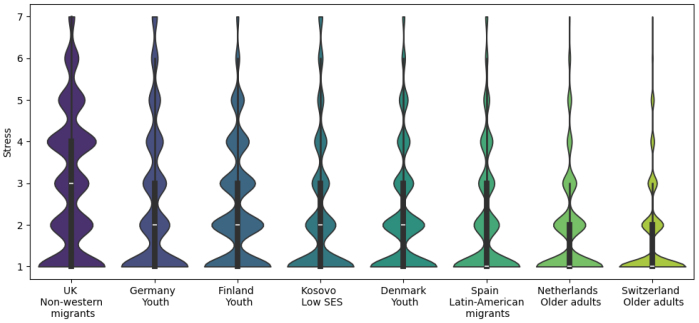
Distribution stress values over countries.

After the preprocessing steps, the dataset comprises 41885 timepoints from 1958 participants. Statistics after preprocessing are shown in Table E.1 in Appendix E. Notably, the proportion of stress transitions varies across countries, with the Dutch sample of older adults (14.51%) and the Swiss sample of older adults (16.91%) having a relatively low percentage of transitioning to elevated stress, indicating relatively stable stress values. In contrast, the UK sample of non-western migrants aged 18 and older shows the highest percentage of transitioning to elevated stress (25.08%). Additionally, Fig. 2, representing a scaled violin plot such that violins have the same width, shows that the Swiss sample consisting of older adults and the Dutch sample consisting of older adults report lower and less spread stress values, whereas the UK sample of non-western migrants aged 18 and older report higher and more widely distributed stress levels. The lower mean stress observed in the Swiss sample of older adults and the Dutch sample of older adults is consistent with previous findings reflecting that stress levels tend to decrease with age (Stefaniak et al., 2021). However, differences between the study populations extend beyond age alone, and other contextual factors (e.g., gender distribution, urbanicity, etc.) may also contribute to the observed variation in stress levels.

#### 4.1.1 Floor effects and the intraclass correlation coefficient

To further evaluate the distribution of stress values, we examined the presence of floor effects, which occur when average scores are low and responses are skewed towards the lower end of the scale, resulting in limited variability (Siepe et al., 2025). Following Siepe et al. (2025), we examined floor effects by determining the proportion of participants who selected the lowest response stress category more than 80% of the time over the full data period, although different thresholds could be used. This criterion was met by only 12.0% of users. Additionally, we examined the distribution of the personal stress thresholds used to classify each observation as normal or elevated stress. This threshold was defined per user per timepoint as the median stress score over the past 7 days, meaning it could vary over time within the same participant. Across all observations in the second week (data on which predictions are made), the threshold was most commonly 1.0 (45.76%), with the occurrence of other thresholds as follows: 2.0 (25.81%), 3.0 (11.97%), 4.0 (10.07%), 5.0 (2.91%), and 6.0 (0.38%). Each of the intermediate threshold values (e.g., 1.5) was observed in fewer than 1% of the observations.

To better understand the ratio of within- and between-individuals variability, we have calculated the intraclass correlation coefficient (ICC) (Siepe et al., 2025). The ICC was 0.44, indicating that 44% of stress variance is due to between-person differences, while the remaining 56% reflects within-person variability. This suggests that a substantial portion of variance is caused by within-person fluctuations rather than differences between individuals.

### 4.2 Model performance on combined country data: All EMA vs stress-only EMA

Table 2 reports the performance of the ML models trained on the combined training sets from all countries and evaluated on the combined test sets. It also reports the mean ROC–AUC and standard deviation from 5-fold cross-validation on the training data. ML models were trained on features derived from all EMA questions or only on features derived from the stress EMA question. Overall, the ML models achieved higher precision and recall for predicting transitions to elevated stress compared to the baseline models. However, the accuracy of the ML models was lower than that of the ‘most frequent’ baseline, which performs well due to the high class imbalance. Notably, including features based on all EMA questions does not improve performance, indicating that EMA burden could be reduced by only using the stress EMA question. Among the ML models, XGBoost achieved a slightly higher cross-validation ROC–AUC score than logistic regression (0.7034 vs 0.6998). Nonetheless, the overlapping confidence intervals indicate that performance differences between the models are small. Subsequent analyses focus on the XGBoost model using features derived from the stress EMA question. This model had an ROC–AUC score of 0.70 (95% CI 0.67–0.71) on the test set.

Table 3 presents the results from the ablation in which the current timepoint’s stress value was not inferred from the most recent past observation when it was missing. In the main analysis, if the current stress value was missing, the most recent stress rating was utilized to determine whether the participant was in a normal or elevated stress state to define the target variable. In both cases, the subsequent stress value had to be available to define the prediction target. This does not affect the calculation of input features for the models. Overall, ROC–AUC scores remain largely consistent across both approaches.

**Table 2:** Performance tuned ML models and baselines.

Model	Precision normal stress	Recall normal stress	Precision elevated stress	Recall elevated stress	Accuracy	ROC–AUC Train (SD)^a^	ROC–AUC Test (95% CI)^b^
*XGBoost Stress EMA*	0.85	0.82	0.34	0.38	74%	0.70 ± (0.00)	0.70 [0.67, 0.71]
*XGBoost All EMA*	0.85	0.83	0.34	0.38	74%	0.70 ± (0.01)	0.70 [0.67, 0.72]

*Random Forest Stress EMA*	0.85	0.75	0.30	0.46	69%	0.68 ± (0.01)	0.68 [0.66, 0.70]
*Random Forest All EMA*	0.86	0.74	0.31	0.49	69%	0.68 ± (0.01)	0.68 [0.66, 0.70]

*Logistic Regression Stress EMA*	0.86	0.93	0.30	0.16	82%	0.70 ± (0.01)	0.70 [0.67, 0.72]
*Logistic Regression All EMA*	0.86	0.94	0.27	0.14	82%	0.67 ± (0.01)	0.68 [0.65, 0.70]

*Baseline Most Frequent*	0.81	1.00	0.00	0.00	81%	0.50 ± (0.00)	0.50 [0.50, 0.50]
*Baseline Stratified*	0.81	0.81	0.19	0.19	69%	0.50 ± (0.00)	0.50 [0.49, 0.51]

a Mean and SD of ROC–AUC from 5-fold cross-validation used during hyperparameter tuning.

b Mean and confidence interval from the test set using 100 bootstrap samples drawn with replacement.

**Fig. G.1:**
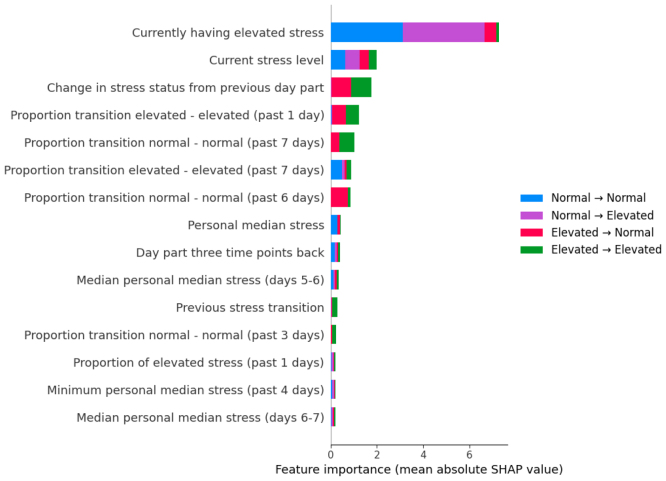
SHAP feature importance: XGBoost model stress EMA question with all possible transition classes.

Finally, an ablation was performed in which all possible stress-state transitions are considered as target classes, including (1) transitions from normal to normal stress, (2) transitions from normal to elevated stress, (3) transitions from elevated to elevated stress, and (4) transitions from elevated to normal stress. As in the alternative analysis described above, missing stress values at the current timepoint were not derived from the most recent stress measurement. Since extending the ROC–AUC score to four classes requires averaging pairwise AUCs, reducing interpretability, the macro-averaged F1 was used for both hyperparameter tuning and evaluation. The ablation was conducted on the XGBoost model using only stress-question features, and class weights were not tuned given the multi-class formulation. Results are shown in Table F.1 in Appendix F. For predictions from normal to elevated stress, a lower recall can be observed compared to Table 2 and 3. However, this could be addressed through oversampling, as XGBoost does not support class weighing in the multi-class setting. SHAP feature importance for the model is reported in Fig. G.1 in Appendix G.

**Table 3:** Performance of tuned ML models and baselines without inferring missing current stress from the most recent measurement.

Model	Precision normal stress	Recall normal stress	Precision elevated stress	Recall elevated stress	Accuracy	ROC–AUC Train (SD)^a^	ROC–AUC Test (95% CI)^b^
*XGBoost Stress EMA*	0.86	0.77	0.33	0.49	71%	0.71 ± (0.01)	0.70 [0.67, 0.72]
*XGBoost All EMA*	0.85	0.84	0.37	0.39	76%	0.71 ± (0.01)	0.71 [0.68, 0.72]

*Random Forest Stress EMA*	0.86	0.75	0.31	0.49	70%	0.69 ± (0.01)	0.69 [0.66, 0.70]
*Random Forest All EMA*	0.86	0.76	0.32	0.49	70%	0.69 ± (0.01)	0.69 [0.66, 0.70]

*Logistic Regression Stress EMA*	0.87	0.94	0.32	0.17	83%	0.73 ± (0.01)	0.71 [0.69, 0.73]
*Logistic Regression All EMA*	0.87	0.94	0.31	0.15	83%	0.71 ± (0.01)	0.68 [0.65, 0.70]

*Baseline Most Frequent*	0.81	1.00	0.00	0.00	81%	0.50 ± (0.00)	0.50 [0.50, 0.50]
*Baseline Stratified*	0.81	0.81	0.20	0.20	69%	0.50 ± (0.00)	0.50 [0.49, 0.51]

a Mean and SD of ROC–AUC from 5-fold cross-validation used during hyperparameter tuning.

b Mean and confidence interval from the test set using 100 bootstrap samples drawn with replacement.

### 4.3 Performance across time slots and personal stress thresholds

Fig. 3 shows ROC–AUC scores for predictions made for different EMA time slots throughout the day and for varying personalized stress thresholds. Fig. 3(a) indicates that model performance is relatively stable across prediction time slots. Only for the prediction for time slot 1 a slightly lower ROC–AUC score can be observed. A possible explanation is that information from the previous day, used to generate summary statistics, may be less precise for predictions made earlier in the day. Nevertheless, the overlapping confidence intervals require cautious interpretation of this observation.

Regarding performance across personalized stress thresholds, Fig. 3(b) shows lower ROC–AUC scores for higher personalized median thresholds, while predictions for threshold 1 achieve the highest ROC–AUC. This may be due to the higher prevalence of threshold 1 in the sample (45.76%), which is also reflected in the narrower confidence interval.

Fig. 3ROC–AUC Scores by prediction time slot and personalized stress threshold.Fig. 3

**(a):**
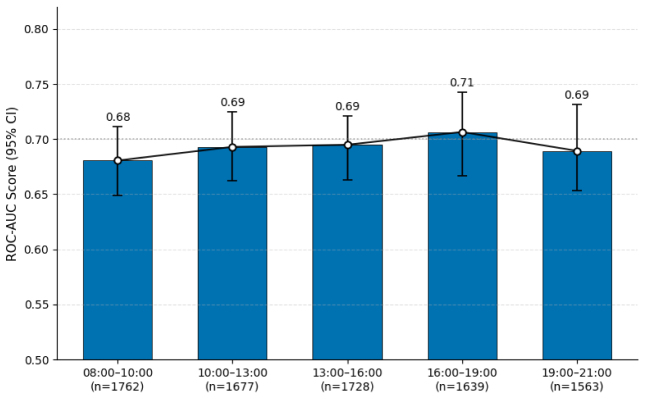
ROC–AUC by Prediction Time Slot.

**(b):**
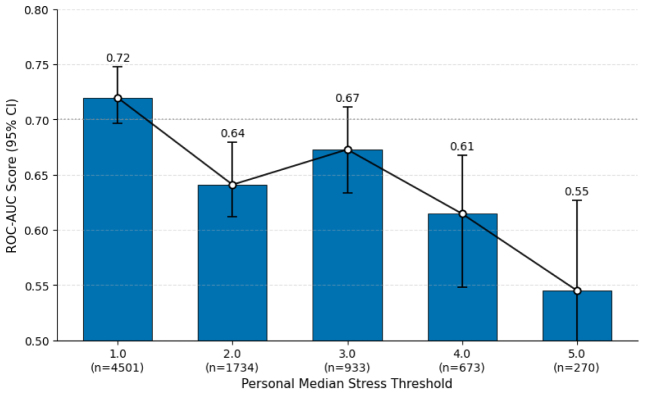
ROC–AUC Across Personal Stress Thresholds.

### 4.4 Performance of global vs. Country-specific models

**Fig. 4:**
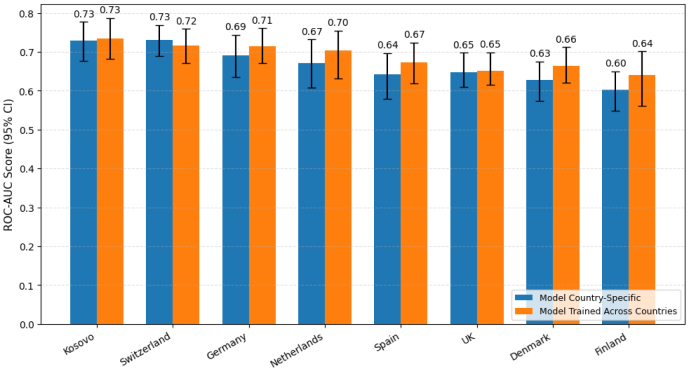
Performance models trained per country vs. model trained over all Countries: ROC–AUC scores.

Fig. 4 presents the macro ROC–AUC scores per country’s test set for two model configurations: (1) a model trained on training data from all countries, and (2) models trained separately for each country. Overall, we can observe that the model trained on the training sets of all countries has a slightly higher performance than the models trained per country. This could be caused by the larger dataset size, suggesting that additional data can help capture more robust patterns. Moreover, performance of the global model is relatively stable across the countries, indicating that the globally trained model generalizes well over the countries.

### 4.5 Model transferability of country-specific models

**Fig. 5:**
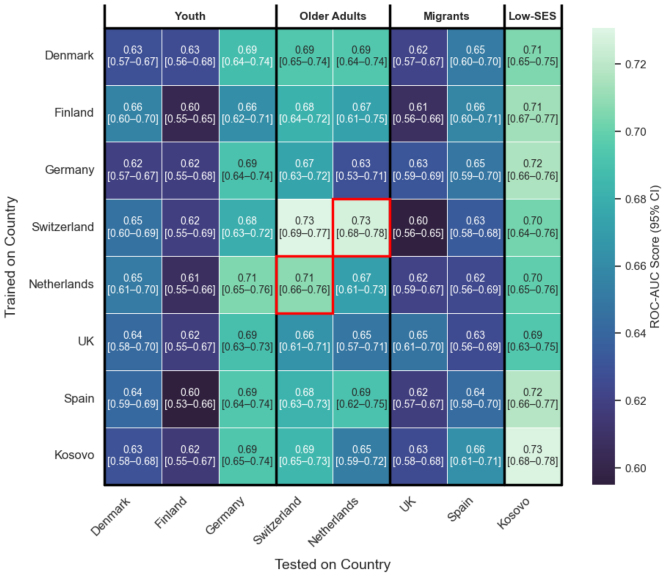
Model transferability of country-specific models: Cross-country-and-population ROC–AUC scores.

Fig. 5 displays a heatmap of the ROC–AUC performance for ML models trained on the training data of one country and applied on the test set of another country. This shows cross-country-and-population performance. Countries are grouped by similar target populations, and the diagonal entries from top left to bottom right correspond to the left blue bars in Fig. 4, representing performance when training and testing on the same country. Overall, cross-country-cross-population deployment did not substantially reduce performance compared to the country-specific models, suggesting that models trained on data from one population can often be effectively applied to another. In some cases, models trained on data from one country achieved better performance on other country’s datasets. This may be explained by the smaller size of individual country-specific training datasets compared with models trained on pooled data from all countries, which is also reflected in the largely overlapping confidence intervals. Notably, strong cross-country-cross-population performance was observed between the Swiss and Dutch populations of older adults. Models trained on the Swiss sample performed well when tested on the Dutch sample, and vice versa. This finding may be explained by similarities between these populations in terms of mean stress levels and the proportion of transitions to elevated stress.

### 4.6 Explainability

**Fig. 6:**
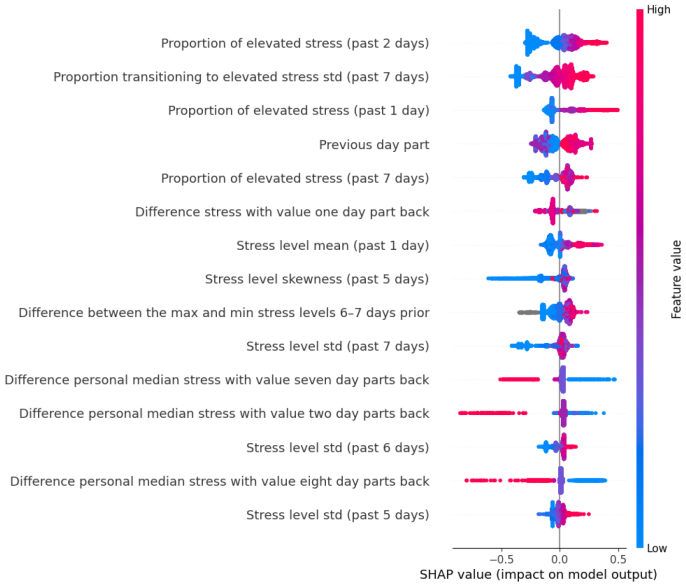
SHAP feature importance: XGBoost model stress EMA question.

In Fig. 6, the SHAP plot is shown for the XGBoost ML model trained only on the features derived from the stress-related EMA question. The plot illustrates how the 15 most influential features (out of 30 in total) contribute to the models’ predictions, ranked from highest to lowest impact. Feature ‘Proportion of elevated stress (past 2 days)‘, reflecting how often a person had elevated stress over the past 2 days, has a high impact. Higher values of this feature increase the probability of predicting a transition to an elevated stress state, as indicated by the positive SHAP values for higher values of this feature. Additionally, feature ‘Proportion transitioning to elevated stress std (past 7 days)‘, indicating the standard deviation of the proportion of transitions to elevated stress over the past 7 days, increases the probability of predicting a transition to an elevated stress state.

## 5 Discussion

This study examined the feasibility of using EMA data in combination with ML to forecast transitions from normal to elevated stress, with the goal of supporting timely and personalized intervention delivery, such as delivering a stress intervention or a notification. Our results show that ML models can reasonably well predict a transition to elevated stress, achieving an ROC–AUC of 0.70, compared to 0.50 for a classifier that returns random predictions. Notably, the model trained across all populations performed comparable to population-specific models, suggesting that a single ML model could be trained over all populations, reducing complexity. Additionally, models utilizing only features related to the stress EMA question performed comparable to models trained on all EMA questions, indicating that collecting only the stress EMA item may be sufficient for stress forecasting while reducing participant’s EMA burden. Finally, validation across countries indicated that country-specific models performed in general well across most populations.

Despite the ROC–AUC score of 0.70, performance metrics precision and recall suggest that the model was more effective at recognizing stability in normal stress than at predicting transitions to elevated stress states. This may be caused by class imbalance in the dataset, which we addressed by tuning the weight assigned to the positive class. The best performing model had a recall of 0.38 and a precision of 0.34, meaning that the model identified 38% of true transitions to elevated stress, while 34% of its predictions of transitions to elevated stress were correct. This has implications for real-world applications: in a notification system, such performance would result in both missed cases and false alarms. However, the classification threshold along the precision–recall curve can be adjusted to better suit different priorities: it can be set to minimize false alarms at the cost of missing more true transitions, or to maximize the detection of true transitions to elevated stress at the cost of more false alarms.

Concerning EMA burden reduction, it could be observed that including EMA items beyond the stress question did not improve predictive performance, suggesting that additional EMA items do not include additional information over the stress EMA question. In other words, these items may measure constructs related to stress, but less accurate than the stress EMA itself. Alternatively, the features derived from the EMA question may not be optimal; different summary statistics or aggregation strategies might further exploit their information. However, a broad range of summary statistics was already applied, including trend-based features calculated by subtracting timepoints. Another possible explanation is that the use of the seven-day median stress threshold could affect the potential contribution of additional EMA items. Nevertheless, this personalized threshold has practical value, as it enables tailored, actionable intervention triggering.

Regarding generalizability, training a model on the combined training datasets and testing on each countries’ test set, did not decrease performance compared to country-specific models, indicating that the model generalizes well across countries and can handle differing stress distributions. A similar pattern was shown in cross-country-cross-population transfer experiments, where a model trained on one country’s data and tested on data of another country, generally did not result in reduced performance. However, since populations differed by both country and target group, it is unclear whether differences stemmed from country or population characteristics. In some cases, a model trained on one country had a higher ROC–AUC score on the dataset of another country, likely because individual country training sets are smaller than the pooled dataset. This is also reflected in the overlapping confidence intervals.

Finally, SHAP feature importance analysis showed that the most influential predictor of forecasting transitions to elevated stress was the proportion of elevated stress over the past two days. This aligns with findings from Siepe et al. (2025) who reported temporal dependencies in EMA-based stress assessments, indicating that stress states are autocorrelated and tend to persist over time. However, the interpretation of feature importance should be considered in the context of the prediction task, as the model was specifically designed to forecast transitions from normal to elevated stress rather than stress states more broadly.

Compared to previous studies on predicting stress from EMA data (Li et al., 2022, Ng et al., 2022), our approach differs in a key way. Rather than using predetermined thresholds or predicting exact stress values, we defined normal and elevated stress relative to each individual’s recent stress distribution. This approach allows for personalized intervention delivery by taking into consideration individual variability in stress perception (Harris et al., 2023). Additionally, we evaluated model performance across countries, providing insights into the generalizability of results and the potential for cross-population deployment. Moreover, other studies commonly focus on detection of stress with ML (Khan et al., 2024, Lisowska et al., 2021, Sarker et al., 2017), instead of forecasting future stress values.

This study also knows some limitations. First, the study period was only two weeks. EMA adherence is known to decline over weeks (Tonkin et al., 2023), therefore, utilizing data from longer time-periods could give better insights about predictive performance over longer periods. Second, normal and elevated stress were defined using a seven-day rolling window. This approach was chosen to reduce the influence of outliers and to prevent fluctuations caused by time-of-day and weekday effects (Siepe et al., 2025). However, the threshold may still have been affected by periods of unusually high stress due to specific life events. Nonetheless, using a longer period to define baseline stress may introduce other confounding factors, such as distal and seasonal effects (e.g., holidays or starting a new job) (Smyth et al., 2023). In addition, longer-term baselines may be less flexible, as more recent stress measurements contribute relatively little to the classification of current stress states (Smyth et al., 2023). Third, floor effects resulting from limited variability in EMA data and a skewed response distribution may have affected the results (Siepe et al., 2025). It could be observed that the mean stress level across countries was approximately 2 on a 1–7 scale, reflecting an overall low stress level. However, only 12.0% of the participants reported a stress rating of 1 more than 80% of the time, which does not suggest a strong flooring effect. Additionally, the personal median threshold used to define normal and elevated stress was skewed towards a value of 1 (45.76% of observations), which likely contributed to the relatively higher ROC–AUC for threshold 1. Nevertheless, no strong performance decrease was observed for thresholds 2 and 3, indicating that the model generalizes reasonably well to higher threshold values. However, lower performance was observed for personal stress thresholds above 3. Fourth, we only focused on making predictions for the next timepoint. In future work, it could be investigated how predictions on different time windows (e.g. next 24 h) affect performance. Fifth, it was assumed that the data were Missing At Random (MAR). However, Missing Not At Random (MNAR) mechanisms cannot be excluded. If missingness was systematically related to unobserved stress levels, the results may have been affected by this. Sixth, individual characteristics may influence EMA adherence (Murray et al., 2023). Incorporating passive measurements from wearable devices, such as smartwatches, could enable continuous stress monitoring and reduce reliance on self-reports. However, passive sensor data, such as from cardiovascular measures, may not always accurately reflect self-reported stress measures (Vaessen et al., 2021). Finally, ML models were not applied in real-life intervention delivery. As a next step, we plan to integrate the algorithm into a mental health app to trigger intervention recommendations when elevated stress is predicted.

**Table A.1:** EMA questions.

Question	Scale

Questions at All 5 Timepoints	

Q1: At the moment I feel sad	1–7
Q2: At the moment I feel bored	1–7
Q3: At the moment I feel stressed	1–7
Q4: At the moment I feel irritable	1–7
Q5: At the moment I feel joyful	1–7
Q6: At the moment I feel relaxed	1–7
Q7: At the moment I feel energetic	1–7
Q8: At the moment I feel enthusiastic	1–7

Morning Questions	

Q9: I slept well last night	1–5
Q10: I expect today to be a stressful experience	1–7
Q11: I am confident I can cope with today’s challenges	1–7
Q12: I am worried about how today will turn out	1–7

Afternoon (3 Timepoints) + Evening Questions	

Q13: Since the last questionnaire I felt overwhelmed by emotions	1–7
Q14: Since the last questionnaire I implemented strategies to feel better (e.g., talking with a friend, have a walk, meditation, etc.)	1–7
Q15: Since the last questionnaire I had enough skills or resources to cope with daily stressors and challenges	1–7
Q16: Since the last questionnaire I acted according to my values and beliefs	1–7
Q17: Since the last questionnaire I have been worrying	1–7
Q18: Did you experience a stressful event since the last prompt?	yes/no

Evening Questions	

Q19: Today, I have been physically active	1–7
Q20: Today, my day has been...	1–7
Q21: Completing the questionnaires was burdensome	1–7
Q22: I received social support today	1–4

## 6 Conclusion

Stress can have a significant impact on mental health, contributing to conditions such as anxiety, depression, and substance abuse (Konstantopoulou et al., 2020, Sinha et al., 2024). Given these effects, it is important to explore effective strategies for monitoring and managing stress. mHealth technologies, combined with ecological momentary assessment data and machine learning, offer a promising framework for tracking and predicting stress in daily life. In this study, we formulated the stress prediction task as a binary classification problem, focusing on predicting transitions from normal stress to elevated stress in each individual, an approach that enables insights for intervention delivery. Furthermore, we used each individual’s stress distribution to further personalize predictions. Our results demonstrated that ML models can predict transitions more accurately than baseline predictors such as a random predictor, and that models trained across all vulnerable populations achieve performance comparable to models trained per population. Furthermore, feature importance analysis showed that often having elevated stress over the past two days was an important predictor. Finally, cross-country evaluations indicated that population-specific models generalized well across most populations. Future research should examine, through real-world experimental studies, whether delivering stress-related interventions based on EMA data and machine learning predictions can lead to meaningful improvements in well-being.

## CRediT authorship contribution statement

**Rutger van der Linden:** Conceptualization, Software, Formal analysis, Investigation, Writing – original draft, Writing – review & editing, Visualization. **Diana Burychka:** Writing – review & editing. **Asmae Doukani:** Writing – review & editing. **Gonçalo Gonçalves:** Data curation, Resources, Writing – review & editing. **Eline Henrotte:** Project administration, Writing – review & editing. **Rocio Herrero:** Writing – review & editing. **Milena Imwinkelried:** Project administration, Writing – review & editing. **Elona Krasniqi:** Writing – review & editing. **Samuel Lam:** Writing – review & editing. **Lisa Groenberg Riisager:** Writing – review & editing. **Kathrin Schopf:** Writing – review & editing. **Claire Rosalie van Genugten:** Writing – review & editing. **Minja Westerlund:** Writing – review & editing. **Rosa Baños:** Funding acquisition, Project administration, Writing – review & editing. **Arlinda Cerga Pashoja:** Funding acquisition, Project administration, Writing – review & editing. **Naim Fanaj:** Funding acquisition, Project administration, Writing – review & editing. **Tobias Krieger:** Funding acquisition, Project administration, Writing – review & editing. **Kim Mathiasen:** Funding acquisition, Project administration, Writing – review & editing. **Artur Rocha:** Funding acquisition, Project administration, Data curation, Resources, Writing – review & editing. **Silvia Schneider:** Funding acquisition, Project administration, Writing – review & editing. **Andre Sourander:** Funding acquisition, Project administration, Writing – review & editing. **Annet Kleiboer:** Funding acquisition, Project administration, Writing – review & editing. **Mark Hoogendoorn:** Conceptualization, Funding acquisition, Project administration, Supervision, Writing – review & editing. **Aneta Lisowska:** Conceptualization, Supervision, Writing – review & editing.

## Declaration of Generative AI and AI-assisted technologies in the writing process

During the preparation of this work, the author(s) used ChatGPT (OpenAI) to rephrase texts. After using this tool/service, the author(s) reviewed and edited the content as needed and take(s) full responsibility for the content of the publication.

## Funding

This work was supported by the European Union’s Horizon Europe research and innovation programme under grant number 101081020. The content of this article reflects only the authors’ views and do not necessarily reflect those of the European Union or the European Health and Digital Executive Agency (HADEA). Neither the European Union nor the granting authority can be held responsible for them.

## Declaration of competing interest

The authors declare that they have no known competing financial interests or personal relationships that could have appeared to influence the work reported in this paper.

**Data availability**

Data may be made available on request. The study is registered in the Open Science Framework (OSF) in which meta-data is shared: https://doi.org/10.17605/OSF.IO/KTJ7Q.

## Figures and Tables

**Table B.1:** Recruitment procedure and participant reimbursement by country.

Country	Recruitment	Reimbursement
Denmark	Open recruitment strategy using posters at relevant educational institutions and social media posts.	Participants who completed the baseline assessment received a voucher of 200 DKK. If they completed between 50%–70% of the EMA measurements, they received 300 DKK, and if they completed at least 90%, they received 400 DKK in the form of a voucher.
Finland	Open recruitment strategy through distribution of posters and ads (in print and digital form) at relevant educational institutions and educational institution platforms, and Instagram posts.	Participants who completed the baseline assessment and at least 50% of the EMA measurements throughout the study period received a €50 compensation.
Germany	Open recuritment strategy, mainly using the distribution of flyers (in print and digital form), within schools and youth recreation and sports clubs.	Participants received a financial compensation of a maximum of €40 in the form of a voucher for completing at least 70% of all assessments (<50% €20; 51%–69% €30).
Kosovo	Open recruitment strategy through distribution of the posters and informative flyers in community centers, social work institutions, university libraries, NGO focused on mental health, LinkedIn, Twitter, landing page, as well as Bluesky.	Participants who completed the baseline assessment, and at least 80% of EMA measures were reimbursed for €30.
The Netherlands	Open recruitment strategy in 4 main cities (Amsterdam, The Hague, Rotterdam & Utrecht) in the Netherlands. Recruitment through community centers, social workers, public libraries, GPs, shops, local newspapers, local news websites, social media and Facebook & Instagram ads.	Participants who completed the baseline assessment received €20,-. For completing the EMA measures participants could receive up to €30,- dependent on their adherence rate ( 15% -> €5; 30% -> €10; 45% -> €15; 60% -> €20; 75% -> €25; 90% -> €30)
United Kingdom	Open recruitment strategy through a website landing page, community workers, social media, university platforms, newsletters, and local cultural centers.	Participants who completed all baseline questionnaires and at least 70% of the EMA measurements received a £30 One4all voucher. Those who completed all questionnaires but fewer than 70% of EMA measurements received a £20 One4all voucher
Spain	Open recruitment strategy through community centers, social workers, and mental health services, as well as social media and the word-of-mouth.	Participants who completed the baseline assessment and at least 70% of the EMA measurements received a €50 voucher.
Switzerland	Open recruitment through community centers, social workers, mental health services, senior universities, the general public, and word of mouth.	Participants who completed the baseline assessment and at least 80% of the EMA measurements received a CHF 50 voucher.

**Table D.1:** Values hyperparameter Grid XGBoost.

Hyperparameters	Values
n_estimators	100, 300
learning_rate	0.1, 0.01
max_depth	3, 7, 9
subsample	0.9, 1.0
scale_pos_weight	2.5, 3.0
num_features	20, 30

**Table D.2:** Values hyperparameter grid random forest.

Hyperparameters	Values
n_estimators	100, 300
max_depth	3, 7, 9
min_samples_split	2, 5
min_samples_leaf	1, 2
class_weight	2.5, 3.0
num_features	20, 30

**Table D.3:** Values hyperparameter grid logistic regression.

Hyperparameters	Values
C	0.001, 0.01, 0.1, 1
class_weight	2.5, 3.0
solver	saga, sag
max_iter	50, 100, 200
num_features	20, 30

**Table E.1:** Population statistics after preprocessing.

Country	Group	Participants	Timepoints	Transition to stress
*Denmark*	Youth	290	5802	1197 (20.63%)
*Finland*	Youth	210	4172	856 (20.52%)
*Germany*	Youth	265	5401	1105 (20.46%)
*Kosovo*	Low SES	269	6204	1021 (16.46%)
*Netherlands*	Older adults	134	3046	442 (14.51%)
*Spain*	Latin American migrants	209	4143	808 (19.50%)
*Switzerland*	Older adults	333	7934	1342 (16.91%)
*UK*	Non-western migrants	248	5183	1300 (25.08%)

**Total**	–	**1958**	**41 885**	**8305 (19.27%)**

**Table F.1:** Performance of the stress-only XGBoost model for predicting all possible stress-state transitions classes.

Transition class	Precision	Recall	F1-Score	Support
Normal → Normal	0.83	0.97	0.90	8125
Normal → Elevated	0.39	0.08	0.13	1747
Elevated → Elevated	0.56	0.49	0.53	1517
Elevated → Normal	0.61	0.68	0.64	1770

*Overall Performance*				
Accuracy	76%			
Train Macro F1 (SD)^a^	0.53±0.00			
Test Macro F1 (95% CI)^b^	0.55[0.54,0.56]			

a Mean and SD of macro F1 across cross-validation training splits.

b Mean and 95% CI estimated via bootstrap resampling of the test set.

## Appendix A EMA questions asked to participants

See Table A.1.

## Appendix B Recruitment and reimbursement

See Table B.1.

## Appendix C Formulas features

### C.1 Elevated stress

Binary feature showing whether the current timepoint reflects normal or elevated stress, based on median stress past 7 days.

(C.1) $${\text{Elevated stress}}_{t}=\left\{\begin{array}{cc} 0,\; & \text{if}Stress_{t}\leq \mathrm{median}\left(Stress_{\left[t-7\text{days}\right]}\right)\\ 1,\; & \text{if}Stress_{t}>\mathrm{median}\left(Stress_{\left[t-7\text{days}\right]}\right) \end{array}\right)$$

### C.2 Proportion of elevated stress

Feature indicating proportion of timpepoints with elevated stress over the past d days.

(C.2) $${\text{Prop. elevated stress}}_{d}=\frac{\text{\# timepoints elevated stress past}d\text{days}}{\text{\# timepoints past}d\text{days}}$$

### C.3 Proportion transitioning to elevated stress

Feature indicating proportion of transitions to elevated stress over the past d days. This feature is only calculated based on timepoints where a user had normal stress, as transitions to elevated stress could only occur from these states.

(C.3) $${\text{Prop. transitioning to elevated stress}}_{d}=\frac{\text{\# transitions to elevated stress past}d\text{days}}{\text{\# timepoints past}d\text{days normal stress}}$$

## Appendix D Hyperparameters

### D.1 Hyperparameter grid XGBoost

See Table D.1.

### D.2 Hyperparameter grid random forest

See Table D.2.

### D.3 Hyperparameter grid logistic regression

See Table D.3.

## Appendix E Statistics per country after preprocessing

See Table E.1.

## Appendix F Performance stress-only XGBoost model over all possible transition classes

See Table F.1.

## Appendix G SHAP feature importance stress-only XGBoost model with all possible transition classes

See Fig. G.1.
